# Characteristics and treatment outcomes of pan-urothelial cell carcinoma: a descriptive analysis of 45 patients

**DOI:** 10.1038/srep18014

**Published:** 2015-12-10

**Authors:** Dong Fang, Pei Liu, Xuesong Li, Gengyan Xiong, Lei Zhang, Nirmish Singla, Guangzhi Zhao, Qun He, Zhisong He, Liqun Zhou

**Affiliations:** 1 Department of Urology, Peking University First Hospital, Institute of Urology, Peking University, National Urological Cancer Centre, Beijing, China; 2 Department of Urology, University of Texas Southwestern Medical Center, Dallas, TX, US

## Abstract

The incidence of pan-urothelial cell carcinoma (panUCC), which refers to the presence of both bilateral (UTUC) and bladder tumor (BT), is relatively low. However, the profile of a panUCC cohort of patients remains to be elucidated. We reviewed the data of consecutive UTUC patients who received treatment at our center from 1999 to 2012. Overall, 45 patients were included in this study, with a median age of 64.5 years. Fourteen patients initially presented with unilateral UTUC, 11 initially with BT, and the remainder with multiple tumors. Patients with UTUC were more likely to manifest higher rates of muscle invasion and larger-sized tumors. Five patients were treated with complete urinary tract exenteration (CUTE), and most patients (73.3%) received combined management with conservative and radical surgery. After a median follow-up of 77 months, 18 patients (40%) died including 15 (33.3%) due to cancer. Higher tumor stage was the only risk factor predictive of worse survival. Nineteen patients experienced local recurrence after conservative surgery. This study indicated that PanUCC involves either synchronous or metachronous presentation of tumors with a high risk of tumor recurrence, progression, and dissemination after conservative surgery.

Urothelial carcinomas are distinguished by their ability to develop multiple foci in a synchronous or sequential fashion throughout the urinary tract^1^. Upper tract urothelial carcinoma (UTUC) and bladder tumor (BT) often co-exist^2^,^3^, and 1.6% to 6.9% of UTUC patients have been reported to suffer from synchronous or metachronous bilateral disease^4^,^5^,^6^.

Several case reports found patients with both bilateral UTUC and BT^7^,^8^,^9^,^10^. Nguyen *et al.* proposed the term pan-urothelial urothelial cell carcinoma (panUCC) to describe cancer in both upper urinary tracts and the bladder^11^. They analyzed 36 patients treated between 1988 and 2013 in two American centers and found that pan-urothelial involvement indicates poor prognosis^11^.

Given the rarity of UTUC^12^, the incidence of panUCC is relatively low, and the characteristics of this cohort of patients remain to be elucidated. Whether complete urinary tract exenteration (CUTE), which entails radical removal of both kidneys and bladder, should be the standard surgical option for these patients remains controversial. Further, considering the heterogeneity and differences in incidence of urothelial carcinoma between Asian and Western patients^13^, profiling the disease in an Asian cohort may facilitate management and elucidation of the underlying mechanisms. In an attempt to address these issues, we performed a descriptive analysis of clinical and pathological features along with surgical outcomes of patients with panUCC in a large Chinese center.

## Results

### Clinical characteristics, initial presentation and tumor progression

Following approval by the institutional review board, we reviewed the data of consecutive UTUC patients who received treatment in the Department of Urology, Peking University First Hospital from 1999 to 2012. Inclusion criteria entailed diagnosis of urothelial carcinoma in the bilateral upper tracts and in the bladder either synchronously or metachronously. For patients who did not undergo surgical intervention, we defined tumor presence by positive urine cytology in conjunction with direct visualization of the tumors via endoscopy or conclusive imaging studies. A total of 45 patients qualified for our study based on these criteria.

The median age of these urban patients was 64.5 years (IQR 58–70), with 15 male (33.3%) and 30 female patients (66.7%). Four patients (8.9%) had a personal history of another malignancy. Only one patient had a family history of malignant tumors. Twenty-one patients consumed Chinese herbs containing aristolochic acid (AA) for at least six months; 9 patients denied the intake of such herbs, and the remaining patients were unable to provide any data regarding their herbal treatment. Six patients reported a history of renal transplantation due to severe chronic kidney disease (CKD), 12 patients suffered from end-stage renal disease (ESRD), and 7 were on dialysis. Patient characteristics are summarized in Table 1.

Fourteen patients initially presented with unilateral UTUC, 11 with BT, and the remainder with tumors at multiple synchronous sites: 10 with bilateral UTUC, 6 with unilateral UTUC plus concomitant BT, and 4 with synchronous panUCC. The median time from initial presentation to tumor dissemination to a second or a third site was 8 months (range 0–84) and 21 months (range 0–141), respectively. In patients who initially presented with tumor in only one site, the median time for metachronous tumor involvement at second and third sites, was 24 months (range 3–84 and 3–141, respectively).

Pathological features of the first tumor site and third (last) metachronous tumor site are listed in Table 2. Patients who initially presented with synchronous tumor in all 3 sites were excluded from this analysis, as were patients with incomplete data. Although no differences in tumor stage or grade were seen between the first and third tumors, the third tumor site more frequently displayed sessile architecture and multiple foci.

### Location-based characteristics

Clinicopathologic features stratified by initial site of tumor involvement (i.e., upper tract, bladder, or both) are displayed in Table 3. Initial presentation involved 48 upper tract tumors and 21 bladder tumors. Location of initial tumor had no effect on the time to development of metachronous tumors or on final tumor stage or grade.

**Figure 1:**
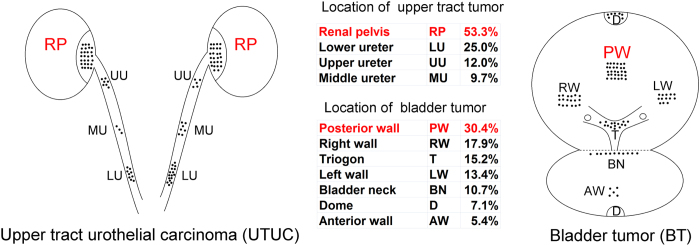
Location of panUCC in upper tract and bladder. Note the number of black dots only reflects percentage.

We also compared the pathological characteristics of all tumors located in the upper tracts and in the bladder regardless of the sequence of presentation. More muscle-invasive (T ≥ 2) and larger-sized tumors were characteristic of UTUC, while BTs more often featured multiple foci (Table 4). The location of all UTUC and BTs based on available data is shown in Fig. 1. Tumors were ubiquitous in the urothelium, though the posterior wall of the bladder was the most frequent tumor location, while tumors in the upper tract of renal pelvis were more common than ureteral tumors.

### Treatment and prognosis

As shown in Table 1, five patients were treated with CUTE, while most patients (73.3%) received combined conservative and radical surgery. Three patients received conservative surgery in all the three sites. Four patients with metachronous UTUC following unilateral treatment refused contralateral radical nephroureterectomy (RNU) despite clinician recommendations. They were treated with unilateral RNU (in 1 case) or nephron-sparing techniques (1 case of pelvic tumor resection and 2 cases of endoscopic ablation) plus synchronous or metachronous transurethral resection (TUR).

**Figure 2:**
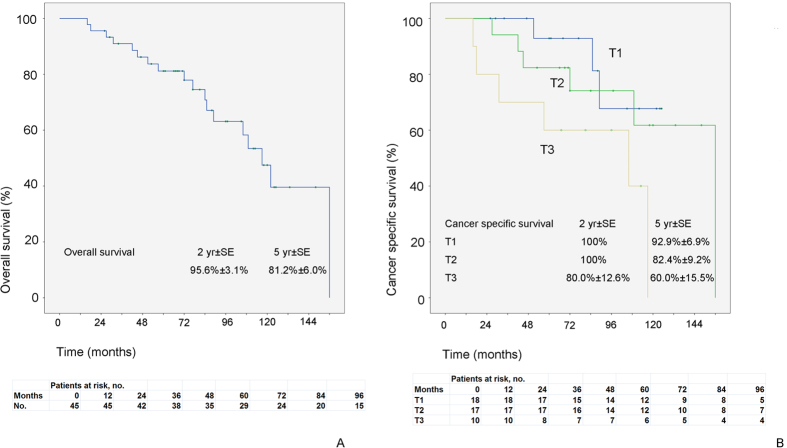
Estimated Kaplan-Meier curve representing overall survival (a) and cancer-specific survival curves stratified by tumor stage (b).

The median follow-up was 77 (16–156) months. Eighteen patients (40%) died, including 15 (33.3%) from urothelial cancer. The 2- and 5-year cancer-specific survival (CSS) rates were 95.6% and 81.2%, respectively. Univariate analysis revealed that higher tumor stage was the only risk factor contributing to worse CSS (Fig. 2), while no parameter had a statistically significant impact on overall survival (Table 5). Neither location of initial tumor nor surgical approach independently predicted survival outcomes.

As shown in Table 4, a large proportion of UTUC cases were treated with radical surgery (RNU), while most BT patients underwent conservative surgery (TUR). Nineteen patients (with 22 sites) who received conservative surgery experienced local recurrence. Following surgery for recurrent tumor, 10 patients ultimately underwent CUTE. During follow-up, 19 patients required permanent dialysis (none of whom were renal transplant recipients), including 6 patients with at least one kidney.

## Discussion

To the best of our knowledge, this is the first study detailing the clinicopathologic characteristics and treatment outcomes of panUCC in a Chinese cohort, with further substratified analysis based on tumor location. Elucidating the features of this uncommon disease entity, our data may contribute to optimal and personalized risk-based therapy while supporting future investigations into the biological mechanisms underlying multiple urothelial tumors. In our previous study, women tended to more often suffer from synchronous bilateral UTUC^5^, while patients with CKD had higher risk for synchronous and metachronous bilateral UTUC^4^,^5^ as well as concomitant non muscle invasive bladder cancer (NMIBC) with unilateral UTUC^2^. We have previously found that women also tended to consume more AA-containing herbs^2^,^4^,^5^,^14^. The current study confirms the elevated risk for panUCC in women and in patients with renal insufficiency. Unlike the cohort of Nguyen *et al.*^11^, these patients do not manifest other distinguishing features, such as history of other malignant tumors or tobacco use. Generally, considering the prevalence of CKD and female gender among Chinese patients with UTUC^14^, it is difficult to recognize a patient who is at risk of pan-urothelial recurrence based on initial presentation alone.

Although both UTUC and BT affect urothelial tissues, there are significant differences in their mechanisms of carcinogenesis, biological behaviors and prognosis^15^,^16^. In panUCC, regardless of synchronous or metachronous involvement, tumors in different locations still manifest the biological characteristics of primary UTUC and primary BT. In our cohort, BT demonstrated higher rates of multiple foci, while upper tract tumors were frequently muscle-invasive and larger. TUR is a commonly used bladder-preserving strategy especially for early stage tumors, while the current gold-standard treatment for UTUC remains RNU. Compared with Nguyen *et al.*’s study^11^, more patients that initially presented with UTUC than with BT (48 and 21, respectively, vs. 19 and 17, respectively), and 20 patients (44.4%) initially presented synchronously with two or three sites of tumor involvement. Given the higher prevalence of UTUC in China, especially in patients with CKD, endemic differences between the cohorts may potentially account for this discrepancy.

Tumors that metachronously originated at the third site more frequently demonstrated sessile architecture and multiple tumor foci when compared with tumors at the initial site. This finding was consistent with Nguyen *et al.*’s findings, which showed that six patients who presented with low-grade disease were upgraded at the time of recurrence^11^. This seems to suggest that recurrent tumors tend to progress and demonstrate more aggressive characteristics, which emphasizes the significance of radical surgery such as CUTE. Although no consensus has been reached on the role of tumor burden in oncologic control^3^,^17^, our results suggest that recurrent disease might be lethal, underscoring the poor prognosis of panUCC. Tumor stage was the only predictive factor for worse prognosis in our survival analysis, although limited by small sample size.

Previous studies recommend CUTE for panUCC patients, especially those with ESRD^7^,^8^,^9^,^10^,^18^,^19^. Patients with severe CKD or dialysis have been shown to manifest a higher risk of developing urothelial carcinoma^18^,^20^, and of experiencing tumor recurrence^4^,^21^,^22^. Radical surgery decreased the possibility of recurrence and cancer-specific mortality^23^, and especially for severe CKD patients there’s no worry about the preservation of renal function. Even in unilateral UTUC patients, a prophylactic contralateral nephroureterectomy was recommended for uremic patients or renal transplant recipients^24^,^25^. Oncologic control and improved survival should remain the dominant therapeutic goals. Holton *et al.*^19^ also proposed that minimizing recurrence and the need for repeat surgery should be the key goals of intervention, rather than concern about the unacceptably high morbidity of CUTE.

Considering the popularity of TUR for NMIBC and the increasing acceptance of NSS in UTUC^26^,^27^, carefully selected patients with moderate renal function might be amenable to conservative surgery, which may offer improved quality of life, freedom from dialysis, and decreased peri-operative complications, particularly for radical cystectomy versus TUR^27^,^28^. In our study, surgical approach and tumor recurrence exhibited no significant impact on survival, but the high recurrence rate should not be neglected, and it should be noted of the probable tumor progression after recurrence as is discussed above. Besides, 6 patients ultimately required dialysis despite preservation of at least one kidney, suggesting that the renal function deteriorated after frequent surgery. With increased age, the use of organ-conserving surgeries is decreased. Conservative surgeries should be performed following individual risk-benefit analysis and rigorous post-procedure surveillance, while CUTE should remain the standard treatment.

Field cancerization hypothesis^29^ and intraluminal seeding^30^ are currently the two main theories to explain the multifocality of urothelial cancer and recurrence patterns in the urinary tract. In our prior study, we supported the role of field cancerization in explaining this phenomenon^4^,^5^, with the presumption that the high prevalence of CKD and consumption of AA-containing herbs might cause nephrotoxicity and carcinogenicity and result in neoplasms of the urothelial tract. Recurrence patterns in our heterogeneous cohort of panUCC patients include BT after UTUC and UTUC following BT. The two theories certainly co-exist and it would be difficult to claim a dominant hypothesis based solely on clinical information. Molecular biomarker profiling of each lesion in multiple urothelial carcinomas may enable the elucidation of the true relationship with AA.

Our study is limited by its retrospective approach, which precluded evaluation of potentially useful variables such as cytology and lymph node status in all patients. Further, our study cohort might be subject to selection and recall bias. Complete pathological results were not available in some patients. Urethral resection was not performed in every radical cystectomy; however, no positive surgical margin was found and no patients exhibited urethral recurrence during later follow-up. Additionally, indications for second surgery were not standardized and were based on the clinical judgment of the treating physician in conjunction with patients’ preferences and expectations.

## Conclusions

PanUCC presents synchronously or metachronously. It may be difficult to identify patients at risk for metachronous development of PanUCC. We recommend CUTE as the standard surgical intervention for selected PanUCC patients with ESRD. The elevated risk of recurrence, progression, and tumor dissemination following conservative surgery reflect potential disadvantages of organ-conserving strategies.

## Methods

### Patient selection

Ethical approval was obtained from Institutional Review Board of Peking University First Hospital. We reviewed the data of consecutive UTUC patients who received treatment in the Department of Urology, Peking University First Hospital from 1999 to 2012. This research was carried out in accordance with the approved guidelines and informed consent was obtained from all patients. Inclusion criteria entailed diagnosis of urothelial carcinoma in the bilateral upper tracts and in the bladder either synchronously or metachronously. For patients who did not undergo surgery, we defined the presence of tumor as positive urine cytology in conjunction with direct visualization of the tumors by endoscopy or conclusive imaging studies.

### Patient evaluation

All patients were diagnosed with UTUC using computed tomography (CT) or magnetic resonance imaging (MRI), cystoscopy, ultrasound, and/or ureteroscopy with or without biopsy. The estimated glomerular filtration rate (eGFR) was calculated using the modified glomerular filtration rate estimating equation for Chinese patients: eGFR(ml/min/1.73m2) = 175 × Scr^−1.234^ × age^−0.179^ (×0.79 if female)^31^. The tumor stage was assessed according to the 2002 Union for International Cancer Control (UICC) TNM classification. Tumor grading was performed according to the World Health Organization (WHO) classification of 1973. The tumor architecture was defined as papillary or sessile after examining the final specimen.

We defined three separate tumor sites including the left upper and right upper tracts, and bladder. Tumor multifocality was defined as the synchronous presence of two or more pathologically confirmed tumors in a single site. Ureteral tumors were classified as upper ureter (superior to the upper border of the sacrum), middle ureter (between the upper and lower borders of the sacrum) and lower ureter (inferior to the lower border of the sacrum) tumors.

### Treatment

All patients underwent surgery within three months following the development of symptoms. Choice of treatment was left to surgeon’s discretion and performed with patient’s consent. Surgical measures considered in managing UTUC consisted of RNU with resection of bladder cuff and NSS. Treatment for BT included TUR for non-muscle-invasive disease and radical cystectomy for muscle-invasive tumors or superficial disease at high risk for progression. In urethra-sparing radical cystectomy, the surgical margin of the proximal urethra was carefully examined. For patients with synchronous tumor in either 2 or all 3 sites, surgeries for the upper and lower urinary tracts were carried out simultaneously or separately within six months.

A series of post-operative epirubicin or pirarubicin intravesical instillation was administered after TUR showed positive pathologic results, while patients without concomitant BT did not receive post-operative intravesical chemotherapy. None of these patients received neoadjuvant chemotherapy. In some patients, adjuvant chemotherapy or radiotherapy was administered when evidence of distant metastasis or retroperitoneal recurrence was documented.

### Follow-up schedule

Post-operative follow-up consisted of cystoscopy every 3 months for the first 2 years, followed by annual cystoscopy surveillance. Chest X-rays, serum creatinine and abdominal ultrasound or CT/MRI were performed at the same intervals.

In patients treated with organ-preserving surgery, we defined recurrence as urothelial cancer in the same operative site on a subsequent setting, e.g. ipsilateral UTUC after endoscopic ablation or recurrent BT after TUR. Treatment measures for recurrent tumor were left to surgeon’s discretion and performed following patient consent. The cause of death was determined by the patients’ treating physicians or by death certificates. Follow-ups were censored until their last visit or death.

### Statistical analysis

All statistical tests were performed with SPSS 20.0 (IBM Corp, Armonk, NY, USA). Pearson’s test and Chi-square test were used to assess categorical variables, and the Mann-Whitney U test and Kruskal-Wallis H test were used for continuous variables. Univariate and multivariate analyses using the Cox’s proportional hazards regression model were used; only variables that were identified as significant by the univariate analysis were considered for the multivariate analysis. All reported P values were single-sided with statistical significance considered at P < 0.05.

## Additional Information

**How to cite this article**: Fang, D. *et al.* Characteristics and treatment outcomes of pan-urothelial cell carcinoma: a descriptive analysis of 45 patients. *Sci. Rep.* **5**, 18014; doi: 10.1038/srep18014 (2015).

## Figures and Tables

**Table 1:** Patient demographic and histological data.

**Variables**	**N**	
Patients	45	100.0%
Gender		
Male	15	33.3%
Female	30	66.7%
Age		
<70	33	73.3%
≥70	12	26.7%
Preoperative renal function		
CKD1 (eGFR≥90)	2	4.4%
CKD2 (90>eGFR≥60)	14	31.1%
CKD3 (60>eGFR≥30)	13	28.9%
CKD4 (30>eGFR≥15)	4	8.9%
CKD5 (eGFR<15)	12	26.7%
AA-containing herbs		
No	3	6.7%
Yes	21	46.7%
Unknown	21	46.7%
Initial location		
Unilateral upper tract	14	31.1%
Bladder	11	24.4%
Bilateral upper tract	10	22.2%
Unilateral upper tract + bladder	6	13.3%
Bilateral upper tract + bladder	4	8.9%
Initial symptom		
Gross hematuria	38	84.4%
Flank pain	1	2.2%
No symptom	6	13.3%
Diabetes		
Absence	38	84.4%
Presence	7	15.6%
Hypertension		
Absence	30	66.7%
Presence	15	33.3%
Coronary heart disease		
Absence	37	82.2%
Presence	8	17.8%
Cigarette		
Absence	40	88.9%
Presence	5	11.1%
Alcohol		
Absence	43	95.6%
Presence	2	4.4%
Treatment		
CUTE	5	11.1%
Radical combined conservative surgery	33	73.3%
Bilateral RNU+TUR	13	28.9%
RNU+NSS+TUR	19	42.2%
RNU+NSS+cystectomy	1	2.2%
Bilateral NSS+TUR	3	6.7%
Unilateral UTUC untreated	4	8.9%

CKD = chronic kidney disease; eGFR = estimated glomerular filtration rate; CUTE = complete urinary tract exenteration; RNU = radical nephroureterectomy; TUR = transurethral resection; NSS = nephron-sparing surgery.

**Table 2:** Clinical and pathological characteristics between first and last presented tumors^.

	**All**	**Initially presented**	**Last presented**	**Chi-square or Z**	**p value**
**N (%)**	110(100)	57(51.8)	53(48.2)		
**Tumor location, no.(%)**				1.166	0.280
Upper urinary tract	72(65.5)	40(70.2)	32(60.4)		
Bladder	38(34.5)	17(29.8)	21(39.6)		
**Radical resection, no.(%)**				1.943	0.163
Yes	51(46.4)	31(54.4)	20(37.7)		
No	55(50.0)	26(45.6)	29(54.7)		
**Radical resection after surgery for recurrence, no.(%)**				2.888	0.089
Yes	57(51.8)	35(61.4)	22(41.5)		
No	49(44.5)	22(38.6)	27(50.9)		
**Multifocality, no.(%)**				9.448	0.002*
No	55(50.0)	37(64.9)	18(34.0)		
Yes	53(48.2)	20(35.1)	33(62.3)		
**Architecture, no.(%)**				10.388	0.001*
Papillary	93(84.5)	55(96.5)	38(71.7)		
Sessile	8(7.3)	0(0)	8(15.1)		
**Stage, no.(%)**				0.732	0.694
Ta-1	73(66.4)	42(73.7)	31(58.5)		
T2	21(19.1)	10(17.5)	11(20.8)		
T3	8(7.3)	4(7.0)	4(7.5)		
**Grade, no.(%)**				2.990	0.224
G1	4(3.6)	2(3.5)	2(3.8)		
G2	68(61.8)	32(56.1)	36(67.9)		
G3	27(24.5)	18(31.6)	9(17.0)		
**Tumor size, means ± SD**		1.92 ± 1.56	1.57 ± 1.25	−1.294	0.196

^*^Statistically significant.

^^^since tumors in different site could present synchronously, some patients might have tumors of 2 sites presented initially, and some might have 2 sites presented last.

SD = standard deviation.

**Table 3:** Clinical and pathological characteristics between different sites of initial presentation.

	**All**	**UTUC**	**BT**	**UTUC+BT**	**Chi-square or Z**	**p value**
**N (%)**	45(100)	24(53.3)	11(24.4)	10(22.2)		
**Gender, no.(%)**					0.433	0.805
Male	15(33.3)	7(29.2)	4(36.5)	4(40.0)		
Female	30(66.7)	17(70.8)	7(63.6)	6(60.0)		
**Gross hematuria, no.(%)**					2.253	0.324
No	7(15.6)	2(8.3)	3(27.3)	2(20.0)		
Yes	38(84.4)	22(91.7)	8(72.7)	8(80.0)		
**Initial stage, no.(%)**					7.745	0.101
Ta-1	30(66.7)	15(62.5)	10(90.9)	5(50.0)		
T2	9(20.0)	5(20.8)	0(0)	4(40.0)		
T3	5(11.1)	4(16.7)	0(0)	1(10.0)		
**Initial grade, no.(%)**					7.822	0.098
G1	2(4.4)	0(0)	2(18.2)	0(0)		
G2	26(57.8)	15(62.5)	4(36.4)	7(70.0)		
G3	16(35.6)	9(37.5)	4(36.4)	3(30.0)		
**Interval to 2**^**nd**^ **site, means ± SD**		26.93 ± 19.49	32.27 ± 25.11		−0.302	0.763
**Interval to 3**^**rd**^ **site, means ± SD**		44.07 ± 30.78	41.36 ± 40.09	23.00 ± 28.67	0.478	0.788
**Local recurrence, no.(%)**					5.893	0.053
No	26(57.8)	17(70.8)	3(27.3)	6(60.0)		
Yes	19(42.2)	7(29.2)	8(72.7)	4(40.0)		
**Highest stage, no.(%)**					2.003	0.735
T1	18(40.0)	10(41.7)	3(27.3)	5(50.0)		
T2	17(37.8)	8(33.3)	6(54.5)	3(30.0)		
T3	10(22.2)	6(25.0)	2(18.2)	2(20.0)		
**Highest grade, no.(%)**					3.852	0.146
G2	22(48.9)	12(50.0)	3(27.3)	7(70.0)		
G3	23(51.1)	12(50.0)	8(72.7)	3(30.0)		
**Multifocality, no.(%)**					0.218	0.897
No	10(22.2)	5(20.8)	3(27.3)	2(20.0)		
Yes	35(77.8)	19(79.2)	8(72.7)	8(80.0)		
**Architecture, no.(%)**					0.755	0.686
Papillary	37(82.2)	19(79.2)	10(90.9)	8(80.0)		
Sessile	8(17.8)	5(20.8)	1(9.1)	2(20.0)		
**Tumor size, means ± SD**		2.77 ± 1.85	2.33 ± 0.89	2.79 ± 1.62	0.024	0.988

UTUC = upper tract urothelial carcinoma; BT = bladder tumor; SD = standard deviation.

**Table 4:** Clinical and pathological characteristics between Upper tract tumors and Bladder tumors.

	**All**	**UTUC**	**BT**	**Chi-square or Z**	**p value**
**N (%)**	135(100)	90(66.7)	45(33.3)		
**Presented initially, no.(%)**				0.370	0.587
No	67(49.6)	43(47.8)	24(53.3)		
Yes	68(50.4)	47(52.2)	21(46.7)		
**Radical resection, no.(%)**				33.174	< 0.001*
Yes	63(46.7)	57(63.3)	6(13.3)		
No	68(50.4)	29(32.2)	39(86.7)		
**Radical resection after surgery for recurrence, no.(%)**				21.909	< 0.001*
Yes	69(51.1)	58(64.4)	11(24.4)		
No	62(45.9)	28(31.1)	34(75.6)		
**Recurrence after non-radical surgery, no.(%)**				3.143	0.116
No	46(34.1)	23(25.6)	23(51.1)		
Yes	22(16.3)	6(6.7)	16(35.6)		
**Multifocality, no.(%)**				4.106	0.043*
No	68(50.4)	51(56.7)	17(37.8)		
Yes	65(48.1)	38(42.2)	27(60.0)		
**Architecture, no.(%)**				0.320	0.572
Papillary	115(85.2)	79(87.8)	36(80.0)		
Sessile	10(7.4)	6(6.7)	4(8.9)		
**Stage, no.(%)**				14.313	0.001*
Ta-1	88(65.2)	51(56.7)	37(82.2)		
T2	28(20.7)	26(28.9)	2(4.4)		
T3	10(7.4)	9(10.0)	1(2.2)		
**Grade, no.(%)**				4.017	0.134
G1	6(4.4)	2(2.2)	4(8.9)		
G2	84(62.2)	56(62.2)	28(62.2)		
G3	32(23.7)	24(26.7)	8(17.8)		
**Tumor size, means ± SD**		1.92 ± 1.45	1.16 ± 0.95	−3.547	<0.001*

^*^Statistically significant.

UTUC = upper tract urothelial carcinoma; BT = bladder tumor; SD = standard deviation.

**Table 5:** Univariable analysis of predictive factors for worse overall survival and cancer specific survival.

**Variables**	**Overall mortality**			**Cancer-specific mortality**		
	**HR**	**95%CI**	**p value**	**HR**	**95%CI**	**p value**
Gender (women vs men)	0.488	0.187–1.274	0.143	0.591	0.204–1.712	0.333
Age (continous)	1.033	0.981–1.088	0.216	1.031	0.974–1.091	0.299
Preoperative renal function(eGFR, continous)	1.000	0.985–1.016	0.984	1.000	0.984–1.017	0.982
Surgical approach (not CUTE vs CUTE)	0.466	0.132–1.643	0.235	0.385	0.085–1.736	0.214
Adjuvant therapy (presence vs absence)	0.565	0.129–2.479	0.450	0.711	0.159–3.191	0.657
Renal transplant history (presence vs absence)	1.127	0.255–4.976	0.875	1.280	0.285–5.744	0.747
Tumor stage^ (T3 vs T2 vs T1)	1.627	0.855–3.096	0.138	2.054	1.003–4.205	0.049*
Tumor grade^ (G3 vs G2)	0.470	0.176––1.256	0.132	0.512	0.174–1.508	0.224
Initial location (Both vs BT vs UTUC)	0.982	0.527–1.831	0.954	1.105	0.513–2.008	0.967
Local recurrence (presence vs absence)	0.641	0.084–4.905	0.668	0.792	0.271–2.313	0.670
Tumor size^(continous)	1.256	0.966–1.634	0.089	1.123	0.810–1.556	0.487
CIS,	0.835	0.190–3.670	0.812	0.491	0.064–3.678	0.494
Squamous, sarcoma, or glandular Differentiation	0.929	0.302–2.859	0.898	1.235	0.386–3.949	0.722
Multifocality (presence of multiple foci vs absence)	0.892	0.289–2.749	0.842	0.942	0.262–3.386	0.927
Diabetes (presence vs absence)	1.125	0.595–2.126	0.717	1.130	0.563–2.268	0.730
Hypertension (presence vs absence)	1.064	0.991–1.143	0.087	1.065	0.993–1.143	0.078
Coronary heart Disease (presence vs absence)	1.075	0.878–1.316	0.483	1.092	0.887–1.346	0.407
Smoke (presence vs absence)	1.968	0.553–7.006	0.296	1.433	0.316–6.497	0.641
Alcohol (presence vs absence)	1.396	0.182–10.692	0.748	1.621	0.209–12.576	0.644
Architecture (presence of sessile vs absence)	0.236	0.031–1.788	0.162	0.298	0.039–2.290	0.245

^*^Statistically significant.

^^^The highest stage/grade and largest size of the panUCC were used for analysis.

eGFR = estimated glomerular filtration rate; UTUC = upper tract urothelial carcinoma; BT = bladder tumor; CIS = carcinoma *in situ*; HR = Hazard Ratio; CI = confidence interval.
